# MicroRNA‐99a is a novel regulator of KDM6B‐mediated osteogenic differentiation of BMSCs

**DOI:** 10.1111/jcmm.13490

**Published:** 2018-01-29

**Authors:** Yin Tang, Lan Zhang, Tianchi Tu, Yijia Li, Dana Murray, Qisheng Tu, Jake Jinkun Chen

**Affiliations:** ^1^ Division of Oral Biology Tufts University School of Dental Medicine Boston MA USA; ^2^ State Key Laboratory of Oral Disease West China School & Hospital of Stomatology Sichuan University Chengdu Sichuan China; ^3^ Department of Anatomy and Cell Biology Sackler School of Graduate Biomedical Sciences Tufts University School of Medicine Boston MA USA

**Keywords:** miR‐99a, KDM6B, osteogenic differentiation, BMSCs

## Abstract

Skeletal tissue originates from mesenchymal stem cells (MSCs) with differentiation potential into the osteoblast lineage regulated by essential transcriptional and post‐transcriptional mechanisms. Recently, miRNAs and histone modifications have been identified as novel key regulators of osteogenic differentiation of MSCs. Here, we identified miR‐99a and its target lysine (K)‐specific demethylase 6B (KDM6B) gene as novel modulators of osteogenic differentiation of bone mesenchymal stem cells (BMSCs). Microarray profiling and further validation by quantitative real‐time RT‐PCR revealed that miR‐99a was up‐regulated during osteoblastic differentiation of BMSCs, and decreased in differentiated osteoblasts. Transfection of miR‐99a mimics inhibited osteoblastic commitment and differentiation of BMSCs, whereas inhibition of miR‐99a by inhibitors enhances these processes. KDM6B was determined as one of important targets of miR‐99a, which was further confirmed by luciferase assay of 3′‐UTR of KDM6B. Moreover, *HOX* gene level decreased after transfection of miR‐99a mimics in BMSCs, which indicated that KDM6B is a bona fide target of miR‐99a. Furthermore, in a model of *in vivo* bone regeneration, osteoblast‐specific gain‐ and loss‐of‐function experiments performed using cranial bone defects revealed that miR‐99a mimics‐transfected BMSCs reduced bone formation, and conversely, miR‐99a inhibitors‐transfected BMSCs increased *in vivo* bone formation. Tissue‐specific inhibition of miR‐99a may be a potential novel therapeutic approach for enhancing BMSCs‐based bone formation and regeneration.

## Introduction

Adult stem cells function to maintain normal tissue homeostasis and repair injured tissues 1. Bone marrow mesenchymal stem cells (BMSCs) are typical adult multipotent stem cells with self‐renew capabilities and multilineage differentiation potentials including osteogenic, chondrogenic and adipogenic commitment 2, 3. BMSCs possess outstanding promise for skeletal tissue regeneration therapies due to their easy and convenient isolation, immuno‐modulatory capability, as well as ability to trans‐differentiate, and ability to create a microenvironment inside tissue favourable for tissue repair 4. It has been widely recognized that the modulation of osteogenic potential of BMSCs is crucial in the application for skeletal regeneration 5. However, therapeutic utility of BMSCs hinges upon the understanding of molecular mechanisms essential for regulation of their osteogenic differentiation.

While the importance of the transcriptional control of the skeletal differentiation process is well documented, it has become increasingly evident that epigenetic regulation of gene transcription plays an essential role in osteogenesis. In eukaryotes, gene expressions can be regulated at the chromatin level, which provides means to alter gene expression without direct changing DNA nucleotide sequences, thus ‘epigenetic’ 6. Epigenetic alteration is finely controlled and can therefore be either stably maintained and heritable, or reversible depending on the context 7. Epigenetic regulation is achieved through three main mechanisms: DNA methylation, miRNA‐mediated post‐transcriptional regulation and post‐translational histone modifications, which are fundamental for regulating the differentiation of stem cells and cell fate determination 1, 8, 9, 10.

While many target genes of histone modification, miRNAs and DNA methylation are well known, we do not yet fully understand how these mechanisms cooperate and how they may regulate each other in a biological context. During osteogenic differentiation, epigenetic mechanisms may influence reciprocally as all modifications can occur within the chromatin. Although number of previous studies have been devoted to explore the gene regulation by histone modifications and miRNAs during osteogenic differentiation, their co‐ordinated actions have not been comprehensively examined 11.

To date, the roles of histone acetylation and methylation have been the primary focus for understanding the epigenetic contribution towards the regulation of osteogenic differentiation. Histone modifications can occur simultaneously, sequentially or in combination with other epigenetic mechanisms. As more and more miRNAs appear to significantly modulate osteogenic differentiation in MSCs 12, an increased number of miRNAs has been proved to play a key role in regulating histone acetylation by targeting histone deacetylases (HDACs). HDAC4 has been reported to be targeted by miR‐140 and miR‐29b during osteogenic differentiation 13, 14. Similarly, HDAC5 can be post‐transcriptionally repressed by miR‐2861 in differentiating primary mouse osteoblasts 15, 16. However, whether histone demethylation is also regulated by miRNA remains to be elucidated.

Histone demethylases KDM6B has been recently identified to be required for BMSCs‐mediated bone formation. In this study, we demonstrate that miR‐99a functions as a repressive regulator of osteogenic differentiation of BMSCs, and we identified KDM6B as a new target of miR‐99a. The purpose of the present study was to investigate the potential role of miR‐99a in regulating the histone demethylase KDM6B level in BMSCs during osteogenic differentiation and *in vivo* bone regeneration.

## Materials and methods

### Cell culture

C3H10T‐1/2 cells were maintained in Dulbecco's modified Eagle medium (DMEM) with 10% (v/v) foetal bovine serum (FBS) and 1% penicillin/streptomycin. MC3T3‐E1 cells were cultured in alpha minimum essential medium (α‐MEM) with 10% FBS and 1% penicillin/streptomycin. MLO‐A5 cells were cultured in α‐MEM supplemented with 5% iron‐supplemented calf serum (iCS; HyClone Laboratories, Logan, UT, USA), 5% FBS and 1% penicillin/streptomycin 17.

BMSCs were isolated from long bones of 4‐week‐old C57BL/6J mice, then plated and maintained in DMEM with 20% FBS and 1% penicillin/streptomycin 18, 19. Osteogenic differentiation of BMSCs was carried out using osteogenic medium containing 50 mg/ml ascorbic acid (Sigma‐Aldrich, St. Louis, MO, USA), 5 mM β‐glycerophosphate (Sigma‐Aldrich) and 10 nM dexamethasone (Sigma‐Aldrich).

### miRNA microarray analysis

Total RNA including small RNAs was harvested from MC3T3‐E1 and MLO‐A5 cell cultures using the miRNeasy Mini Kit (Qiagen, Valencia, CA, USA). Five μgs of total RNA from each cell line was sent to LC Sciences (Houston, TX, USA) for microarray analysis. Image processing, data extraction and analysis also were performed by LC Sciences according to manufacturer's instructions 17. The resulting heat map was inspected for differentially expressed miRNAs between two cell lines.

### Sequence alignments and microRNA target prediction

miRNA target prediction was carried out using a combination of the following bioinformatics databases and computational algorithms: Target Scan (http://www.targetscan.org/vert_42/), microRNA.org (http://www.microrna.org/microrna/getGeneForm.do), RNA 22 (https://cm.jefferson.edu/rna22/Interactive/) and miRBase Target (http://microrna.sanger.ac.uk/sequences/). miRNA‐mRNA hybridization structures and free energies between microRNA seed sequences and mRNA sequences were determined by RNAhybrid (http://bibiserv.techfak.uni-bielefeld.de/rnahybrid/).

### Luciferase assay

The luciferase reporter plasmids were constructed by ligating the polymerase chain reaction (PCR) fragments of KDM6B 3′ UTR into pmirGLO miRNA luciferase reporter vector (Promega, Madison, WI, USA). Site mutations in KDM6B 3′ UTR fragments were performed using the Quickchange XL Site‐Directed Mutagenesis Kit (Stratagene, La Jolla, CA, USA). Both the wild‐type and mutated fragments were confirmed by digestion of those constructs with SacI and XohI, and all the new reporter constructs were sequenced to confirm correct DNA orientation and sequence (Tufts University Core Facility, Boston, MA, USA).

For transient transfection, cells were cotransfected with the pmirGLO Vector constructs and miRNA oligos using lipofectamine 2000 reagents (Life Technologies, Carlsbad, CA, USA). Twenty‐four hours after transfection, cells were harvested and analysed for luciferase activity with the Dual‐Glo^®^ Luciferase Assay System (Promega) using a luminometer (Lumat LB 9501; EG&G Berthold, Bad Wildbach, Germany). Normalized firefly luciferase activity (firefly luciferase activity/Renilla luciferase activity) for each construct was compared with that of the pmirGLO Vector no‐insert control. For each transfection, luciferase activity was averaged from six replicates.

### Oligonucleotide transfection

miR‐99a mimics (small, double‐stranded, chemically modified RNA molecules designed to specifically mimic endogenous miRNA molecules and enable miRNA functional analysis by up‐regulation of miRNA activity), miR‐99a inhibitors (anti‐miR‐99a, small, single‐stranded, chemically modified RNA molecules designed to specifically bind to and inhibit endogenous miRNA molecules and enable miRNA functional analysis by down‐regulation of miRNA activity), mirVana™ miRNA Mimics Negative Control (NC), and mirVana™ miRNA Inhibitors Negative Control (NC) were synthesized by Life Technology. Oligonucleotide transfection was performed with Lipofectamine 2000 reagents (Life Technologies). Oligonucleotide was transfected into the cells at 40% confluence with final concentration of 50 nM in the transfection system.

### Quantitative real‐time polymerase chain reaction (qRT‐PCR) for mRNA and miRNA analysis

qRT‐PCR assay for mRNA analysis was performed using SYBR Green Mastermix (Affymetrix, Sunnyvale, CA, USA) on a Bio‐Rad iQ5 thermal cycler (Bio‐Rad Laboratories, Portland, ME, USA). The evaluation of relative expression levels was carried out by the comparative cycle threshold (CT) method using housekeeping gene GAPDH as a control. For miRNA expression analysis, total RNA including small RNAs was extracted using the miRNeasy Mini Kit (Qiagen), and cDNA was synthesized using an NCode miRNA First‐Strand cDNA Synthesis Kit (Life Technologies). qRT‐PCR was performed using an NCode Express SYBR GreenER miRNA qRT‐PCR Kit (Life Technologies) on a Bio‐Rad iQ5 thermal cycler. The relative expression level of U6 snRNA was used to normalize miRNA expression in each sample. The sequences of primers used for amplification are listed in Table 1.

**Table 1:** The sequences of the primers for qRT‐PCR in the experiment

Primer	Sequence
miR‐99a	5′‐ CACCCGTAGAACCGACCTTGCG‐3′
U6	Forward: 5′‐CGCTTC GGCAGCACATATAC‐3′
	Reverse: 5′‐TTCACGAATTTGCGTGTCAT‐3′
KDM6B	Forward Primer: 5′‐TGAAGAACGTCAAGTCCATTGTG‐3′
	Reverse Primer: 5′‐TCCCGCTGTACCTGACAGT‐3′
RUNX2	Forward: 5′‐AAC GAT CTG AGA TTT GTG GGC‐3′
	Reverse: 5′‐CCT GCG TGG GAT TTC TTG GTT‐3′
SATB2	Forward: 5′‐AGG CCC AAG GAA TAA TCA AGC‐3′
	Reverse: 5′‐GCG TCA CAA CGT GAT AGA CAT C‐3′
OCN	Forward: 5′‐GCC GGA GTC TGT TCA CTA CC‐3′
	Reverse: 5′‐GCG CTC TGT CTC TCT GAC CT‐3′
Col‐1	Forward Primer: 5′‐AATGGAAGTTCTACTCGCGTAGG‐3′
	Reverse Primer: 5′‐TTCTCGCCTGGTTGACCTTTG‐3′
Hoxa10	Forward: 5′‐ TTCGCCGGAGAAGGACTC‐3′
	Reverse: 5′‐ TCTTTGCTGTGAGCCAGTTG‐3′
Hoxb2	Forward: 5′‐ ATTCGCCTTTTCTACCGGACC ‐3′
	Reverse: 5′‐ GGGCTATCGAGAGAACCCTG ‐3′
Hoxc10	Forward: 5′‐ ATGACATGCCCTCGCAATGTA ‐3′
	Reverse: 5′‐ CCCCGCAGTTGAAGTCACTC ‐3′
Hoxc6	Forward: 5′‐ CAACGTCGCCCTCAATTCCA ‐3′
	Reverse: 5′‐ AGTCGAGTAGATCCGGTTCTG ‐3′
BSP	Forward: 5′‐GAC TTT TGA GTT AGC GGC ACT‐3′
	Reverse: 5′‐CCG CCA GCT CGT TTT CAT C‐3′
GAPDH	Forward: 5′‐AGG TCG GTG TGA ACG GAT TTG‐3′
	Reverse: 5′‐TGT AGA CCA TGT AGT TGA GGT CA‐3′

### Western blot analysis

Whole protein lysates were prepared with RIPA lysis buffer (Santa Cruz Biotechnology, Inc., Santa Cruz, CA, USA) according to the manufacturer's instructions. SDS‐PAGE electrophoresis was performed using Novex 4–20% Tris‐Glycine gels (Life Technologies), and Western blots were performed using 0.45 mm polyvinylidene fluoride membranes (Millipore, Burlington, MA, USA). Primary antibodies, KDM6B (1:1000), β‐actin (1:10,000), were purchased from Cell Signaling Technology (Daners, MA, USA). The secondary antibodies were horseradish peroxidase‐linked goat‐anti‐rabbit IgG (Santa Cruz Biotechnology). Blots were visualized using SuperSignal West Dura Extended Duration Substrate (Thermo Fisher Scientific, Bilerica, MA, USA).

### Alizarin red S staining quantification assay

Cell culture after osteogenic induction was fixed with 4% formaldehyde, incubated with 40 mM alizarin red S (Sigma‐Aldrich) and inspected using a phase microscope. About 10% acetic acid was used to extract the alizarin red S in cell culture, then 10% ammonium hydroxide was used to neutralize the acid. The absorbance of cell culture extractions and alizarin red S standard at 405 nm were read with a plate reader.

### Silk scaffold preparation and cell seeding

The water‐based silk fibrin scaffolds (disk‐shaped, 2 mm diameter and 2 mm thick) were prepared as described previously 20, 21. For cell seeding, cells with or without miRNA transfections were harvested from the culture substratum and concentrated to 2 × 10^7^ cells/ml in serum‐free medium. Then BMSCs were seeded into the silk scaffolds by pipetting 0.5 ml of the cell suspension onto the materials. The BMSCs and Silk Scaffold constructs were incubated overnight to allow *in vitro* cell attachment before implantation in bone defects.

### Animal surgery and calvarial bone defect model

The animal protocols used in this study were in accordance with guidelines by the Institutional Animal Care and Use Committee at Tufts University/Tufts Medical Center (Approved Protocol #B2011‐49).

Calvarial bone defects were performed using 8‐week‐old C57BL/6J male mice. Briefly, surgeries were performed under anaesthesia as previously reported 20, and a 2‐mm‐diameter calvarial critical‐sized defect was created on each side of the calvarial bone using a dental burr attached to a slow‐speed hand piece with minimal invasion of the dura mater. The critical‐sized defects in mice were randomly divided into four groups (*n* = 5 per group), each group receiving implants containing silk scaffold and BMSCs transfected with different miRNA oligonucleotides.

### Micro CT measurement

Twelve weeks after surgery, the morphology of the regenerated bone in calvarial defects was assessed using a micro CT system (μCT‐40, Scanco Medical, Bassersdorf, Switzerland). The micro CT scanning settings were as follows: 1024 × 1024 pixel matrix and 20 mm slice thickness. After scanning, the micro CT images were processed using a nominal threshold value of 300 as previously reported 20, and a three‐dimensional (3D) histomorphometric analysis was performed automatically. The ratio of bone volume to tissue value was used for comparison parameters in this study.

### Histomorphometric analysis and immunohistochemical staining

For histological examination, calvarial samples were fixed in 10% formalin and then decalcified in 10% ethylenediaminetetraacetic acid (pH 7.0) for 2 weeks, followed by dehydration in ethanol and embedment in paraffin wax. Sections with thickness of five μm were cut and mounted on glass slides. Three randomly selected sections from Statistical Analysis of each sample were stained with haematoxylin and eosin (H&E), using protocols as previously described 22. The immunohistochemical staining (IHC) was performed to detect OCN expression using a Histostain SP kit (Life Technologies). H&E and IHC staining were photographed with an OLYMPUS BX53 microscope. Newly formed bone tissue in H&E‐stained sections was quantified in five sections of five different defect specimens for each group at 200× magnification using Image Pro Plus software. Quantification of new bone formation was expressed as a percentage of the total tissue area of the defect. The localization of OCN staining was studied on transverse sections of the cranium as previously described 22. All slides were coded to prevent the introduction of examiner bias.

### Statistical analysis

All results were expressed as means ± S.D. of three or more independent experiments. One‐way analysis of variance with Tukey post hoc test was used to test significance using the software SPSS 13.0 (SPSS Inc., Chicago, IL, USA). Values of *P* < 0.05 were considered statistically significant.

## Results

### miR‐99a expression is significantly down‐regulated during osteogenic differentiation

**Figure 1:**
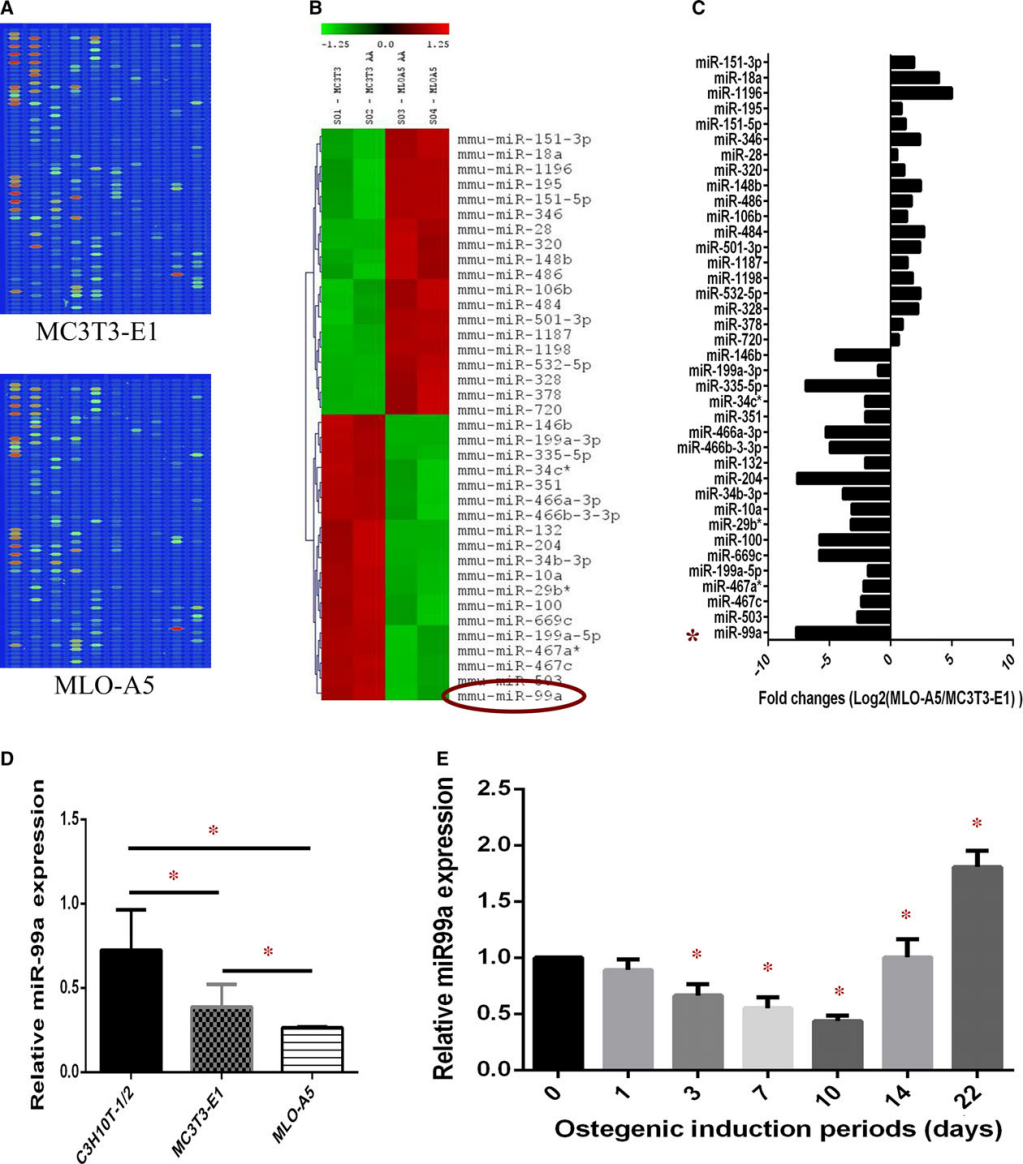
miR‐99a expression is significantly down‐regulated during osteogenic differentiation. (**A**) Original images of the chips. The chip detects miRNA transcripts listed in Sanger miRBase Release 11.0. (**B**) Heat map of miRNAs that are differentially expressed significantly (*P* < 0.05) between MC3T3‐E and MLO‐A5 cells. (**C**) fold differences of 38 differentially expressed miRNAs (*P* < 0.05) between two cell stages. (**D**) qRT‐PCR analysis of the relative miR‐99a expression levels in C3H10T1/2, MC3T3‐E1 and MLO‐A5 cells. (**E**): qRT‐PCR analysis of the relative miR‐99a expression levels in BMSCs during osteogenic induction. Data are shown as mean ± S.D. *P < 0.05.

We first attempted to profile miRNA expression pattern during osteoblastic differentiation. Two well‐studied osteoblastic cell lines, osteoblast‐like cell MC3T3‐E1 and pre‐osteocyte‐like cell MLO‐A5, representing two key developmental stages of the osteoblastic differentiation, were used for miRNA profiling. We isolated total RNA including microRNAs from both cells, and miRNA microarray analysis was performed (Fig. 1A). From a total of 1096 mouse miRNAs assayed, we identified transcripts that were differentially expressed with adjusted *P* values of less than 0.05 between two cell stages. Both MC3T3‐E1 and MLO‐A5 cells exhibit unique and differential miRNA expression profiles (Fig. 1B). Heat maps and fold differences of the differentially expressed miRNAs (Fig. 1B and C) comparisons were produced. We found 38 miRNAs differentially expressed (*P* value < 0.05) between two cell stages. MiR99A changed most dramatically (2^−7.73^ folds), therefore was chosen for further study (Fig. 1B).

We then verified microarray results with qRT‐PCR, found that miR‐99a was significantly down‐regulated in pre‐osteoblast MC3T3‐E1 cells compared to multipotent C3H10T‐1/2 cells, and was further down‐regulated in pre‐osteocyte MLO‐A5 cells (Fig. 1D). We also performed experiments using primary BMSCs, and similar results were obtained. miR‐99a level began to decrease after osteogenic induction, then increase 14 days after osteogenic induction (Fig. 1E).

### KDM6B was predicted to be a target of miR‐99a

miRNA target prediction was carried out using a combination of four computational algorithms. miR‐99a and other four dramatically changed miRNAs (miR‐100, miR‐669c, miR‐34c*, let‐7e) are predicted to potentially target KDM6B during osteogenic differentiation. Therefore, KDM6B was selected for further study as a potential target of miR‐99a (Fig. 3A).

### KDM6B is post‐transcriptionally regulated during osteogenic differentiation

**Figure 2:**
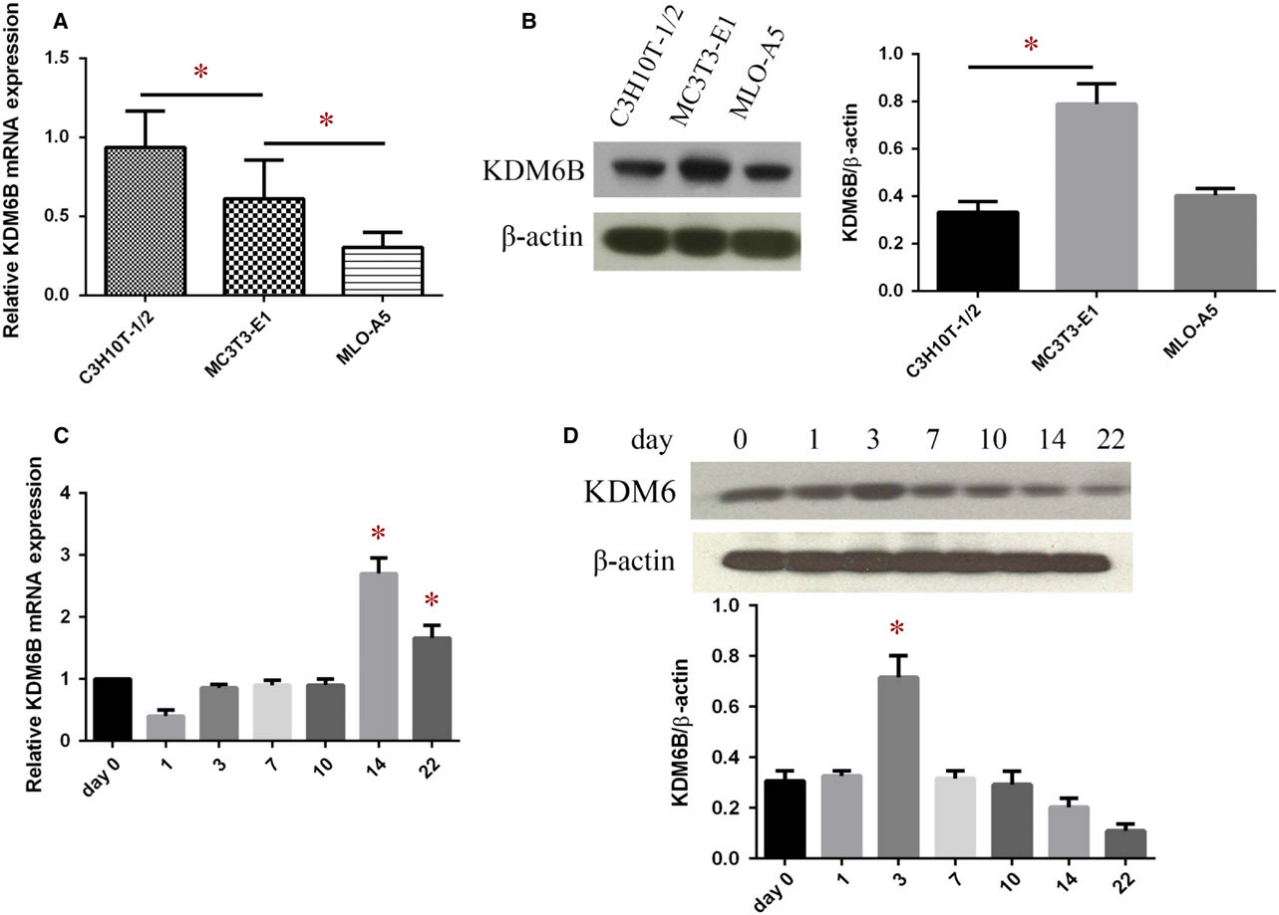
The absence of mRNA‐protein correlation for KDM6B in BMSCs during osteogenic differentiation. (**A**) qRT‐PCR analysis of the relative KDM6B mRNA expression levels in C3H10T1/2, MC3T3‐E1 and MLO‐A5 cells. (**B**) Western blot analysis the KDM6B protein levels in C3H10T1/2, MC3T3‐E1 and MLO‐A5 cells. (**C**) qRT‐PCR analysis of the relative KDM6B mRNA expression levels in BMSCs during osteogenic differentiation. (**D**) Western blot analysis the KDM6B protein levels in BMSCs during osteogenic differentiation. Data are shown as mean ± S.D. *P < 0.05.

*KDM6B* mRNA levels decreased significantly in MC3T3‐E1 cells compared to C3H10T‐1/2 cells and were further down‐regulated in MLO‐A5 cells (Fig. 2A). However, Western blot analysis revealed that KDM6B protein increased more than two folds in MC3T3‐E1 cells compared to C3H10T‐1/2 cells (Fig. 2B).

We also verified these results using primary BMSCs, and similar results were obtained. *KDM6B* mRNA increased significantly at D14 after osteogenic induction (Fig. 2C). Western blot analysis revealed that KDM6B protein level reached the peak on day 3 in BMSCs after osteogenic induction, and then decreased (Fig. 2D).

The absence of mRNA‐protein correlation for KDM6B suggests that the relation between its mRNA and protein is not strictly linear. There are discrepant changes between mRNA and protein levels of KDM6B during osteogenic differentiation. These results clearly indicate that there is post‐transcriptional regulation of KDM6B protein level at certain stages of osteogenic differentiation, and miR‐99a might be involved in this regulation.

### KDM6B expression is specifically regulated by miR‐99a during osteogenic differentiation

**Figure 3:**
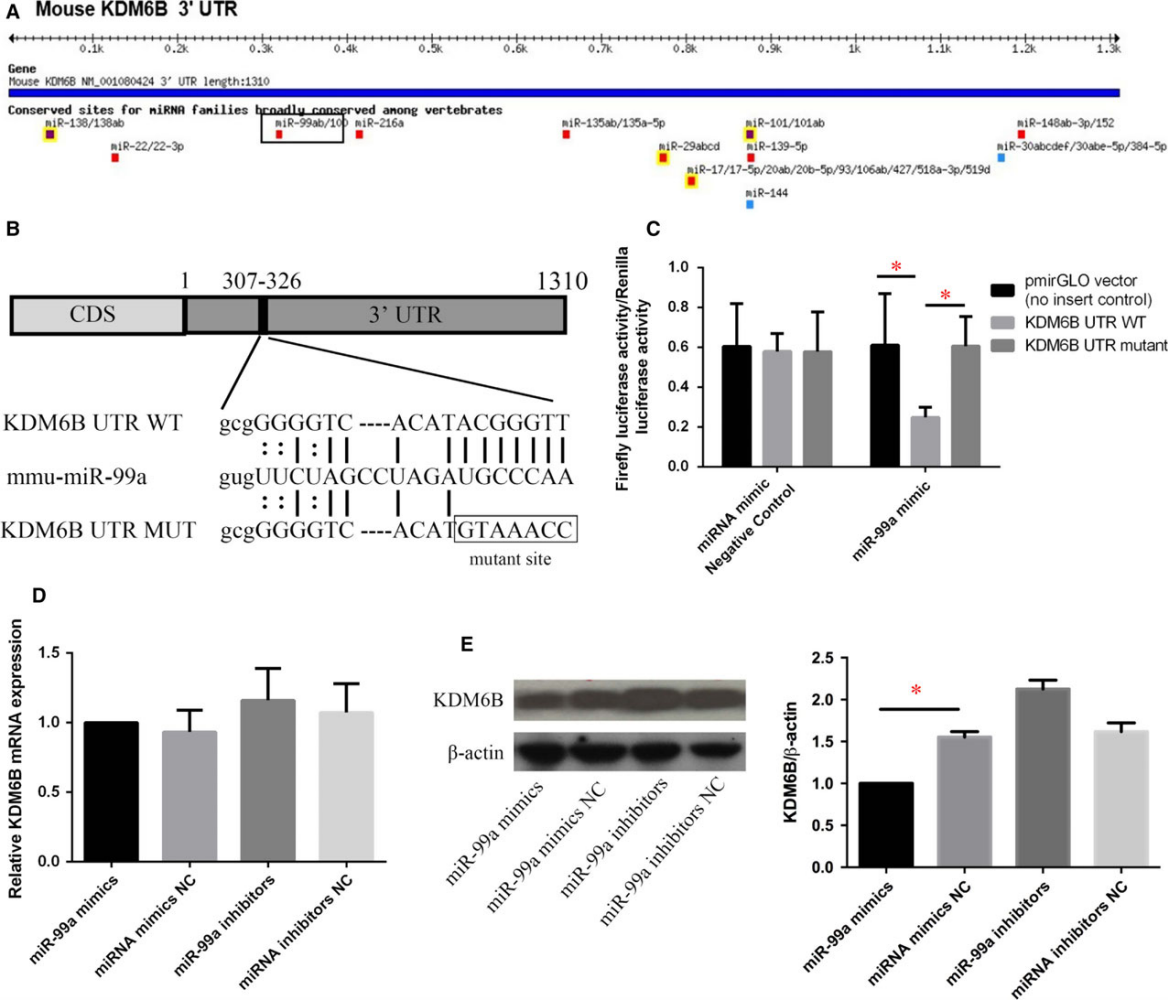
KDM6B expression is specifically regulated by miR‐99a. (**A**) miRNA target prediction using a combination of the following computational algorithms: TargetScan, microRNA.org and miRBase. (**B**) Schema and sequence of predicted miR‐99a binding site on wild‐type (WT) KDM6B UTR, sequence of miR‐99a and sequence of mutated (MUT) KDM6B UTR. (**C**) Wild‐type and mutant KDM6B 3′UTR were, respectively, inserted into a pmirGLO Luciferase Vector, which were transfected into C3H10T‐1/2 cells. Cells were cotransfected with either control or miR‐99a mimic/inhibitor oligomers. miR‐99a effectively suppressed luciferase reporter activity, while the mutations significantly impaired the activity of miR‐99a, relieving repression of luciferase activity. (**D**) qRT‐PCR analysis of the relative KDM6B mRNA expression levels in C3H10T‐1/2 cells. There was no change of KDM6B mRNA expression after miR‐99a mimics transfection. (**E**) Western blotting showed that KDM6B protein level was all significantly down‐regulated by miR‐99a mimics treatment. Data are shown as mean ± S.D. *P < 0.05.

To address whether KDM6B is specifically targeted by miR99a, we performed luciferase assay. We first located the potential seed sequences for miR‐99a at the 3′ UTR of KDM6B mRNA by TargetScan and miRBase (Fig. 3A), and then inserted wild‐type and mutant KDM6B 3′UTR, respectively, into a pmirGLO Luciferase Vector (Fig. 3B). These vectors were transfected into C3H10T‐1/2 cells. Cells were cotransfected with either control or miR‐99a mimics/inhibitors oligonucleotides. We found that miR‐99a mimics significantly suppressed luciferase activity. Furthermore, the mutations effectively reversed the effect of miR‐99a, relieving repression of luciferase activity (Fig. 3C). These results suggest that miR‐99a can directly bind to the 3′UTR of KDMB6, thereby post‐transcriptionally regulating protein levels.

We also detected KDM6B expression after miR‐99a mimics treatment. After miR‐99a mimics transfection, there was no change in *KDM6B* mRNA expression (Fig. 3D). Western blots showed that KDM6B protein level was significantly down‐regulated by miR‐99a mimics treatment (Fig. 3E).

### miR‐99a suppresses osteogenic differentiation of BMSCs

**Figure 4:**
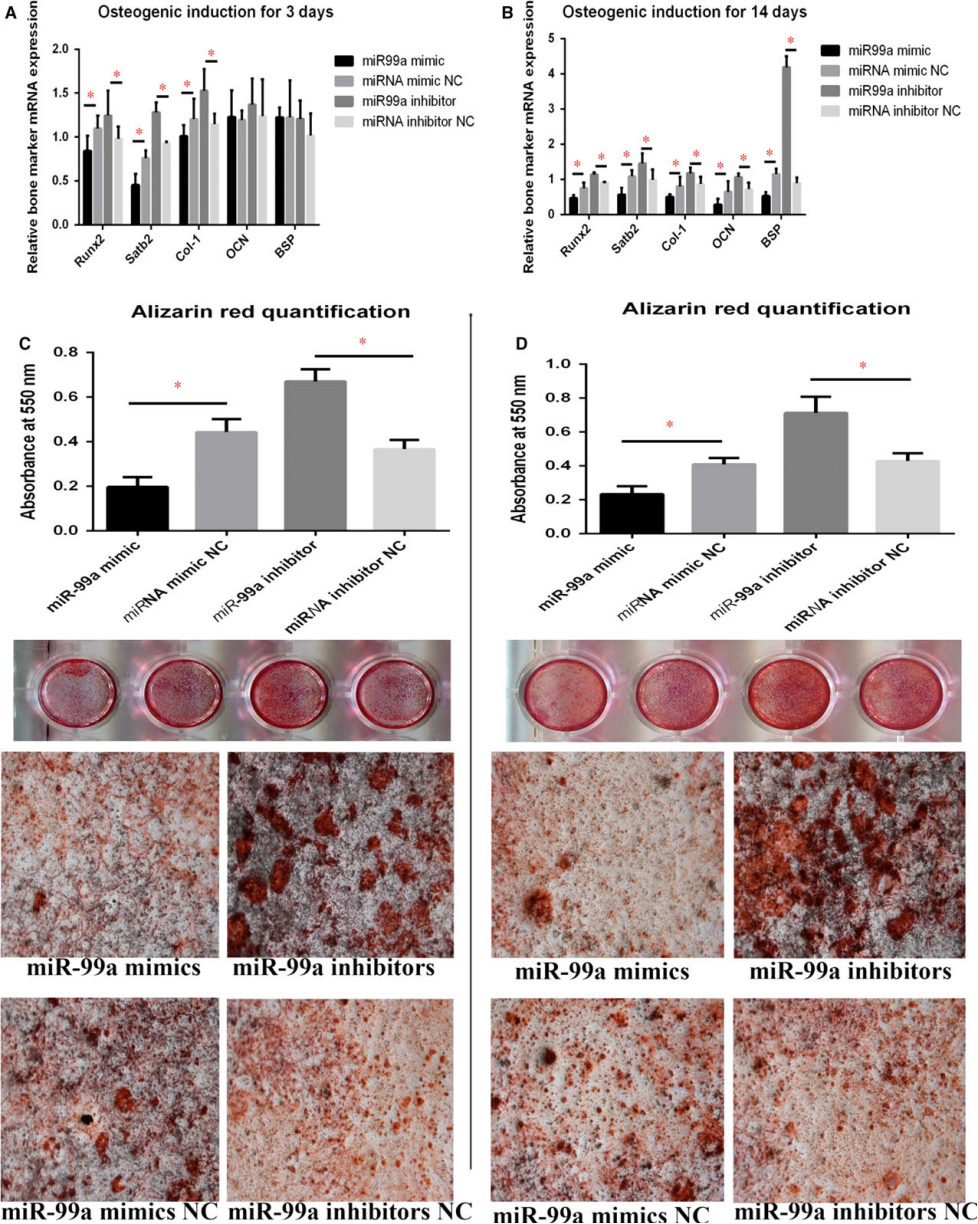
Gain‐ and loss‐of‐function analyses in BMSCs cell cultures suggested that miR‐99a suppresses osteogenic differentiation of BMSCs. (**A**) qRT‐PCR showed that the mRNA levels of Runx2, Satb2, Col‐I were significantly increased by miR‐99a inhibitors after osteogenic induction for 3 days. (**B**) qRT‐PCR showed that the mRNA levels of Osteocalcin (OCN) and Bone sialoprotein (BSP) mRNA levels were also increased by miR‐99a inhibitor after osteogenic induction for 14 days. (**C**) Mineralization of BMSCs was assayed by Alizarin red staining after 21 days of incubation showed that mineralization was significantly reduced in response to miR‐99a mimics in miR‐99a mimics group, compared to negative control groups, while inhibitors group has the increased level of mineralization. (**D**) Mineralization assay was verified using BMSCs incubated for 28 days. The lower two panels of C and D are representative bright‐field micrographs (100×) of the cultures scanned in the upper panels of C and D, respectively. Data are shown as mean ± S.D. *P < 0.05.

We then performed gain‐ and loss‐of‐function analyses in BMSCs cultures. miR‐99a mimics, inhibitors or control oligomers were transfected into BMSCs before osteogenic induction. Bone markers mRNA levels were determined at different time‐points during osteogenic differentiation to evaluate the osteogenic status. qRT‐PCR showed that the mRNA levels of *Runx2*,* Satb2*,* Col‐I* were significantly increased by miR‐99a inhibitors after osteogenic induction for 3 days (Fig. 4A), and also *Osteocalcin (OCN)* and *Bone sialoprotein (BSP)* mRNA levels were also increased by miR‐99a inhibitor after osteogenic induction for 14 days (Fig. 4B). These observations confirmed that miR‐99a mimics inhibited BMSCs differentiation, while miR‐99a inhibitors promoted osteoblastic differentiation of BMSCs.

*In vitro* mineralization of BMSCs was assayed and evaluated by alizarin red staining after 21 and 28 days of osteogenic incubation, respectively, and also showed the same trend. Mineralization was significantly reduced in response to miR‐99a mimics, compared to negative control groups, and was significantly increased in inhibitors group (Fig. 4C and D).

### miR‐99a can regulate *HOX* genes expression in BMSCs by targeting KDM6B

**Figure 5:**
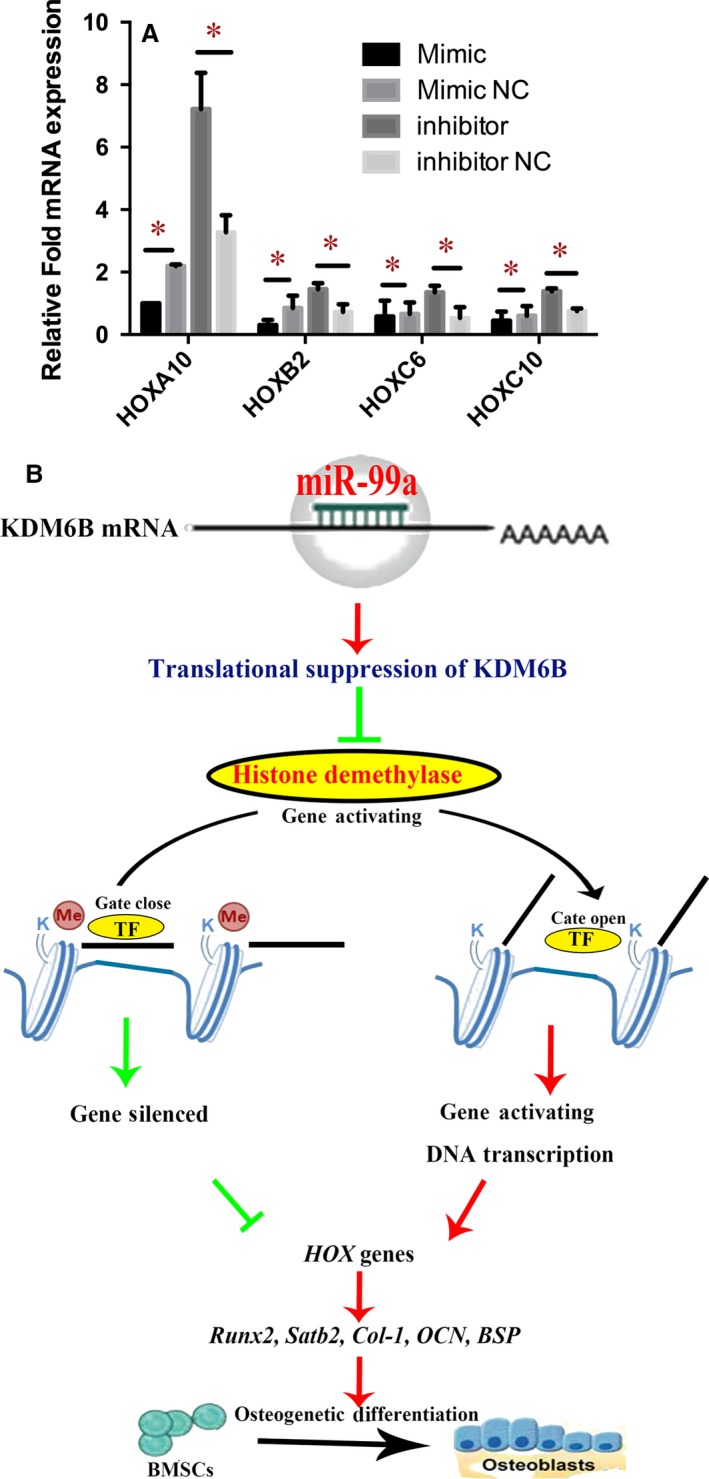
miR‐99a can regulate HOX Genes Expression in BMSCs by Targeting KDM6B. (**A**) qRT‐PCR revealed that the transfection of miR‐99a mimics significantly inhibited the expressions of HOXC6‐1, HOXA10, HOXB2 and HOXC10, while miR‐99a inhibitors can significantly elevate their levels. (**B**) Schematic diagram of mechanism of miR‐99a in osteogenic differentiation of BMSCs. miR‐99a may regulate HOX transcription factors by targeting KDM6B, HOX transcription factors can activate Runx2 transcription and mediate osteoblastogenesis. Thereby miR‐99a may play roles in regulating the osteogenic differentiation of BMSCs. Data are shown as mean ± S.D. *P < 0.05.

It was previously reported that KDM6B regulates the expression of *HOX* genes in embryonic stem cell differentiation through removing the H3K27me3 from histone 23. The epigenetic regulation is spatio‐temporally specific. The loss of H3K27me3 may be KDM6B independent in some cells during specific stages of development 24, but it has been well demonstrated that KDM6B can remove H3K27me3 in MSCs during osteogenic differentiation 4, 23. *HOX* genes encode a large group of homeodomain‐containing transcription factors which are essential in anterior‐posterior axis patterning in embryonic development 25, 26. Moreover, HOX proteins were found to function as transcription factors to activate Runx2 transcription and can also mediate osteogenic differentiation in a Runx2‐independent manner 27. As *HOX* genes are implicated in osteogenic differentiation, we assayed whether transfection of miR‐99a affected the endogenous expression of *HOX* genes. qRT‐PCR revealed that the transfection of miR‐99a mimics significantly inhibited the expressions of *HOXC6‐1*,* HOXA10*,* HOXB2* and *HOXC10*, while miR‐99a inhibitors can significantly elevate their levels (Fig. 5A), implying that miR‐99a might regulate these *HOX* transcription factors by targeting KDM6B, thereby playing roles in regulating the osteogenic differentiation of BMSCs (Fig. 5B).

### miR‐99a is elevated in osteoporotic bone marrow

**Figure 6:**
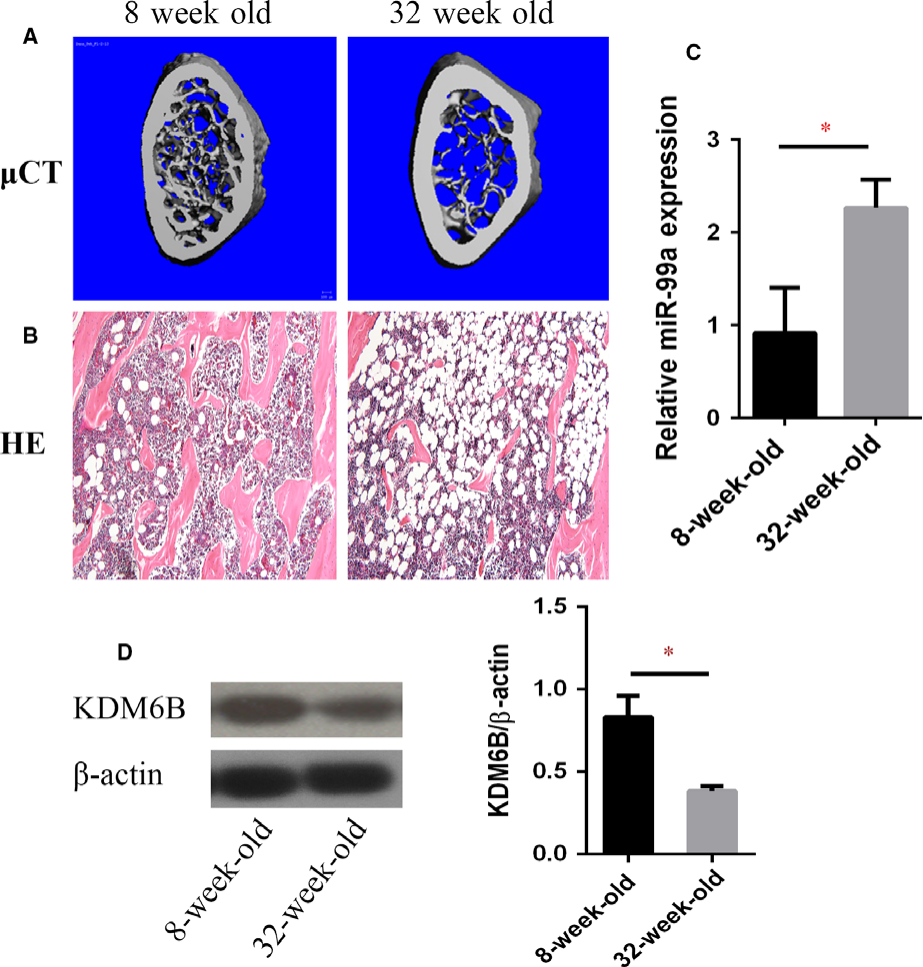
miR‐99a is elevated in Osteoporotic Bone Marrow. (**A**) Representative micro CT images of osteoporotic bone in ageing mice. (**B**) Trabecular bones were decreased, and adipose tissues were increased in ageing mice by histological analysis (HE, ×100). (**C**) qRT‐PCR revealed that the expression of miR‐99a was significantly increased in bone marrow from old mice compared to those from young mice. (**D**) Western blotting revealed that the KDM6B protein was significantly reduced in BMSCs from old mice compared to those from young mice. Data are shown as mean ± S.D. *P < 0.05.

BMSCs in bone marrow can differentiate into osteoblasts, which is required for the maintenance of trabecular bone volume. In ageing mice, osteoblatogenesis is reduced in bone marrow 28. To further confirm that miR‐99a is associated with BMSCs fate commitments, we also examined its status in bone marrow of ageing mice. Compared with younger, 8‐week‐old mice, micro CT showed that the trabecular bone was significantly reduced in the bone marrows of 32‐week‐old mice (Fig. 6A). H&E staining showed that aged femurs had less bone tissues, while more adipose tissue (Fig. 6B). We isolated RNA from bone marrow of both young and old mice. qRT‐PCR revealed that the expression of miR‐99a was significantly increased in bone marrow from old mice compared to those from young mice (Fig. 6C). We also isolated BMSCs from both young and old mice. Western blotting revealed that the KDM6B protein was significantly reduced in BMSCs from old mice compared to those from young mice (Fig. 6D). Our results suggest that the miR‐99a may be a critical epigenetic factor that regulates BMSCs differentiation in bone marrow.

### Bone healing enhanced by inhibition of miR‐99a in BMSCs

**Figure 7:**
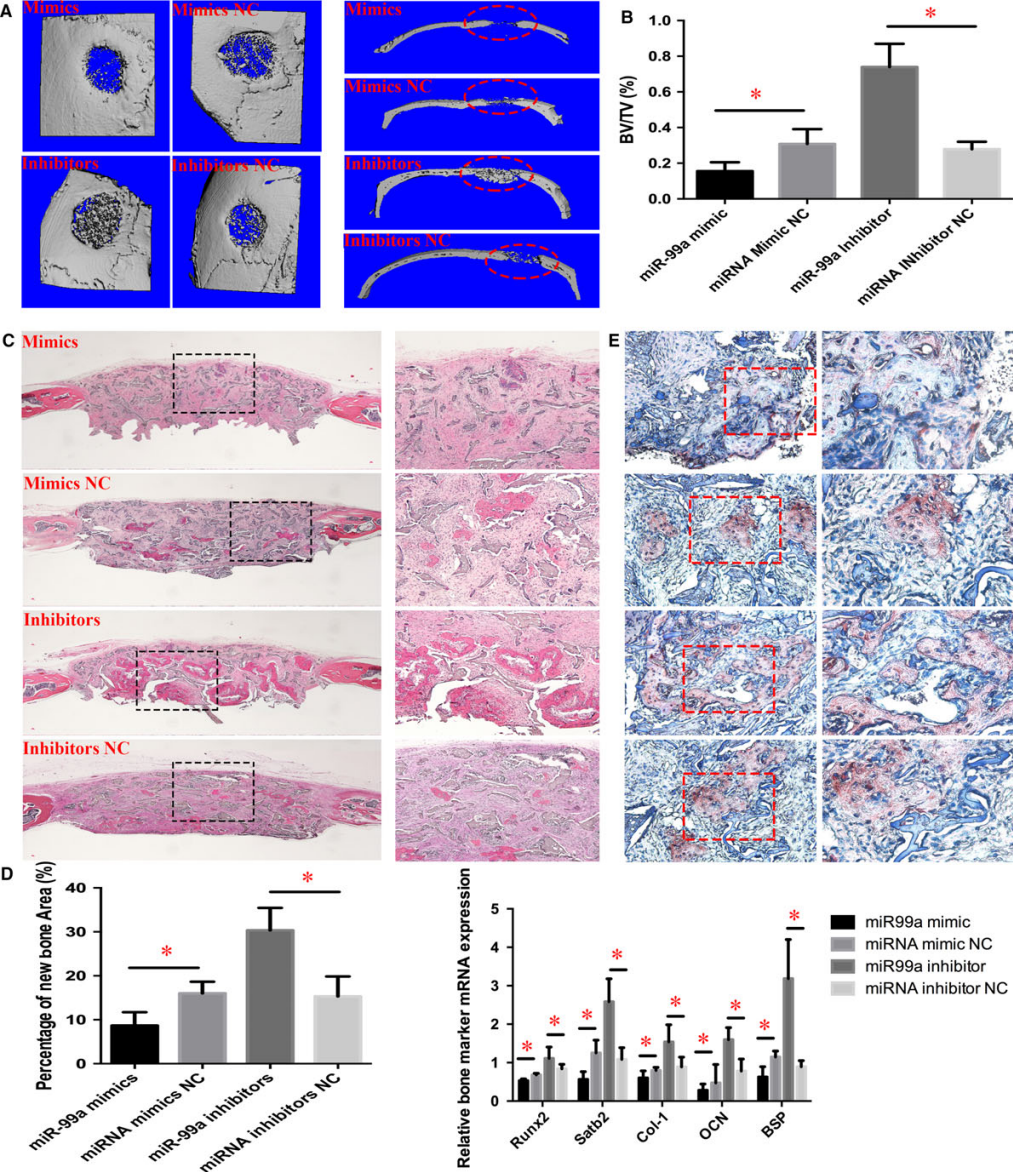
Bone healing enhanced by inhibition of miR‐99a in BMSCs. (**A**) micro CT evaluation showed that more newly formed bone filled the defects of the miR‐99a inhibitors‐modified BMSCs group. Right panel, coronal section of cranial samples. (**B**) Quantification of the new bone regeneration within the calvarial defects. BV/TV was significantly higher for the miR‐99a inhibitors‐modified BMSCs group, and much less bone in miR‐99a mimics‐modified BMSCs group, when compared to NC groups. (**C**) Histological evidence further supported the micro CT results, indicating that the specimens from the miR‐99a inhibitors‐modified BMSCs group showed extensive new bone formation. Left panel, 40×; right panel, 100×. (**D**) Percentage of newly formed bone in different treatment groups measured in H&E staining sections. (**E**) Immunohistochemical analysis of newly formed bone. Left panel, 200×; right panel, 400×. The labels of group images were the same as in (**C**). Stronger OCN expression was observed in the newly formed bone area in the miR‐99a inhibitors‐modified BMSCs group. (**F**) qRT‐PCR analysis of bone marker genes in bone defect tissue. Data are shown as mean ± S.D. *P < 0.05.

Critical size full‐thickness defects of 2 mm in diameter were created in both sides of the calvarial bone and implanted with a silk scaffold seeded with miRNA‐transfected BMSCs. Twelve weeks after surgery, micro CT was performed to detect the morphology of the newly regenerated bone and quantitatively evaluate new bone formation within the defects. More newly formed bone was found inside the defects of the miR‐99a inhibitors‐transfected BMSCs group, when compared to negative control groups, which the miR‐99a mimics‐modified group has decreased bone formation, which showed the formation of scattered new bone in the defect areas (Fig. 7A).

To quantify the new bone formation inside the cranial defects, the ratio of regenerated bone volume (BV) to total defect tissue volume (TV) was measured. BV/TV was significantly higher for the miR‐99a inhibitors‐transfected BMSCs group, while miR‐99a mimics‐transfected BMSCs group has the decreased BV/TV value (Fig. 7B). Histological findings further supported the micro CT results, indicating that the specimens from the miR‐99a inhibitors‐transfected BMSCs group showed the more extensive new bone regeneration and formation (Fig. 7C). In contrast, a small amount of newly formed bone tissue was found in the negative control‐transfected BMSCs, while even less bone in miR‐99a mimics‐transfected group (Fig. 7C). The percentage of newly formed bone area in the miR‐99a inhibitors‐transfected BMSCs group was significantly higher than negative control group (Fig. 7D). To further analyse the new bone regeneration, immunohistochemistry was performed to determine osteocalcin (OCN) expression levels. Immunohistochemical staining exhibited strong expression for OCN in larger areas of new bone inside the defect region from specimens implanted with the miR‐99a inhibitors‐transfected BMSCs, whereas OCN staining was weaker in miR‐99a mimics‐transfected BMSCs (Fig. 7E). Moreover, to further determine the osteogenic effect of miR‐99a inhibitors‐transfected BMSCs in new bone regeneration, total RNA was isolated from defect areas of four groups, and qRT‐PCR was performed to detect mRNA expression levels of *Runx2, Satb2, Col‐1, OCN* and *BSP*. Osteogenic marker mRNA expressions showed a higher level in the defect tissues implanted with miR‐99a inhibitors‐transfected BMSCs group than the negative control group (Fig. 7F), while miR‐99a mimics transected in BMSCs suppressed the expression of these genes. These results demonstrated that BMSCs transfected with miR‐99a inhibitors oligonucleotides enhanced *in vivo* bone healing in mice.

## Discussion

Stem cell regulation in the skeletal system remains relatively unexplored, and numerous attempts have been made to understand their differentiation process under epigenetic regulation. A continuously increasing number of miRNAs have been implicated by previous studies as regulators of different stages of bone developments, osteogenic differentiation and bone regeneration. Due to the complex biology of osteoblastic differentiation and skeletal formation, the number of miRNAs regulating these processes is expected to be large and until now has not been identified in its entirety. In the present study, we identified miR‐99a as a novel regulator of osteogenic differentiation and *in vivo* bone formation of BMSCs. We demonstrated that inhibition of miR‐99a by an inhibitor (anti‐miR) oligonucleotide markedly increased osteogenic differentiation *in vitro* and enhanced *in vivo* bone formation, whereas miR‐99a overexpression reversed these effects. Interestingly, another study has similar results consistent with what we found in our research. They screened miRNA expression profile during MSCs differentiation towards osteoblasts using microarray, and they found several miRNAs including miR‐99a changed dramatically, which suggests that miR‐99a might play a vital role in osteogenic differentiation of BMSCs 29. Several studies have proved that miR‐99a can inhibit tumour cell proliferation and tumorigenesis by directly targeting growth regulator kinase mammalian target of rapamycin (mTOR) in different cancers 30, 31, 32, 33, 34. Moreover, recent studies implicate the mTOR pathway as important in determining MSC fate with ability to induce osteoblast differentiation of MSCs 35, 36, 37. These potential mechanisms suggest that mTOR signalling may also be targeted by miR‐99a in osteogenic differentiation, which needs to be elucidated in the future study.

We selected KDM6B for further study as a potential target of miR‐99a, because at least five dramatically changed miRNAs (miR‐99a, miR‐100, miR‐669c, miR‐34c*, let‐7e) are predicted to potentially target KDM6B during osteogenic differentiation. These findings suggest that KDM6B may play a critical role in osteogenic differentiation, and we confirmed that KDM6B is an important target of miR‐99a during osteogenic differentiation. Both histone modifications and miRNAs play pivotal roles in gene expression regulation, and it has been reported that the mechanisms of miRNA and epigenetic regulation are not entirely separable, and their co‐ordinated actions have not been comprehensively examined 38. Whether KDM6B protein and histone methylation are regulated by miRNAs remains unclear. Our finding for the first time provides a novel investigation into the co‐ordinated actions between histone demethylase KDM6B and miRNA.

Many cell models have been developed to learn osteoblastic differentiation and function. In this study, we utilized two well‐studied osteoblastic cell lines, murine osteoblast‐like cells MC3T3‐E1 and murine pre‐osteocyte‐like cells MLO‐A5, representing two key developmental stages of the osteoblast for miRNA profiling. MC3T3‐E1 was established from normal mouse calvaria, and MLO‐A5 was established from the long bones of 14‐day‐old mouse pups, expressing the large T‐antigen driven by the osteocalcin promoter 39. It may appear to be controversial to compare the miRNA expression profiles between these two kinds of cells line, because they have different origins and backgrounds. To address this concern, we then verified microarray results with RT real‐time PCR using primary BMSCs and obtained similar results with the same trend during osteoblastic differentiation. Suppressed level of miR‐99a is necessary for osteoprecursor cell to differentiate into osteoblasts. When the osteoblasts differentiate further towards the terminal stage of bone‐forming cell, miR‐99a is significantly down‐regulated.

This study used BMSCs to research the relationship of miR‐99a and KDM6B during osteogenic differentiation. Most epigenetic studies are also based on the most‐studied model using MSCs, which can give rise to several cell types, including adipocytes, osteoblasts and chondrocytes 40, 41. However, the existence of MSCs has not yet been proven *in vivo* by fate‐mapping experiments 42. The perisinusoidal MSCs lining bone marrow sinusoids have been regarded as a principal promising candidate for the *in vivo* studies of MSCs. Until most recently, questions have been raised about perisinusoidal MSCs 43. By lineage tracing researches, it has been realized that perisinusoidal lining cells with *in vivo* adipogenic and osteogenic ability differentiate into osteoblast and adipocyte only in mature animals, but neither during bone development nor skeletal growth. Hence, it seems that perisinusoidal MSCs fail to meet those expected criteria of skeletal MSCs. Despite years of intensive research, the existence and specific characteristics of skeletal stem cells remain elusory. Skeletal stem cells should be collectively a group of diverse cells, with different marker patterns and distinct developmental fates, and can be fractionated into subpopulations 44. As a result, a prerequisite for better understanding the genetic and epigenetic controls that guide the osteogenic process is to identify these post‐natal skeletal stem cell populations and to clarify their differentiation potential and cell fate lineages 45, 46.

In this study, to perform gain‐ and loss‐of‐function analyses in BMSCs modified with increased or suppressed levels of miR‐99a, we used mirR‐99a oligonucleotide (mimics/inhibitors) transfection approach. At present, there are two ways to therapeutically manipulate miRNA expression and function 47. Promising tools are those oligonucleotides that mimic or suppress the endogenous mature miRNA by sequence complementarity. Issues to overcome are the efficient delivery and off‐target effects. Naked miRNA oligonucleotides are less efficient due to their *in vitro* or *in vivo* instability because of different nucleases. Lipid‐based vehicles, cationic polymers and viral systems are among the main delivery tools for miRNA‐based strategies. Each of these approaches has its own challenges and still needs improvements to address problems such as cytotoxicity, immunogenicity and low efficiency.

Additionally, the *in vitro* and *in vivo* osteogenic effects of BMSCs transfected with miR‐99a were largely dependent on delivery efficiency and miRNA efficacy in this study. To address this problem, we detected KDM6B protein levels in BMSCs after miR‐99a oligonucleotides transfection. Interestingly, KDM6B protein decreased 34% after mimics transfection, while increased 41% after inhibitors transfection. This is partially in accordant with previous proteomic studies in response to miRNA regulation reported that the average changes in target protein levels are less than two folds following miRNA inhibition 48, 49. This is probably due to the existence of cofactors regulating the activity of miRNAs and a large number of potential mRNA targets. A single miRNA may be potentially related to the repression of hundreds of proteins but this repression is always relatively mild. MiRNA effect is often dependent on stress conditions, whereby both the severity and the type of cellular stress influence whether an mRNA is regulated by a miRNA.

To evaluate the *in vivo* bone formation effect of miR‐99a‐transfected BMSCs, we used critical size cranial bone defect model and silk scaffold in our study. Tissue engineering approach has been widely used in skeletal research field, which is an especially ideal choice to repair larger size bone defects by using resorbable scaffolds supplemented with regeneration‐potential cells and growth factors 50. The distinguishing mechanical properties, biodegradability and low inflammatory response of silk fibroin make it a promising scaffold for bone regeneration 51, 52. Several our previous studies also found that silk fibroin was an excellent scaffold for bone regeneration 18, 53. As a result, we continue using silk fibroin as scaffolds in this study. Besides the scaffolds, the seeded cells and regulators are essential elements for tissue‐engineered bone regeneration. As an important regulator, epigenetic pathways can play a key role in the control of gene expression during different stages of bone development and throughout life, which will lead to more effective ways to prevent and treat bone disease.

There is currently a growing interest in developing novel pharmacological agents based on targeting miRNAs to treat disease, and some of the miRNA inhibitors are undergoing clinical trials 54. Previous studies have shown that targeting miRNA contributed to the development of osteoporosis *in vivo* 55 and led to primary osteoporotic bone in humans 15. We observed that inhibition of miR‐99a in BMSCs enhanced the bone‐forming capacity in cranial bone defects. Thus, inhibiting miR‐99a in BMSCs *in vivo* may lead to enhanced bone regeneration. It is plausible that local implantation of BMSCs deficient in miR‐99a or BMSCs cultured on a functionalized scaffold containing miR‐99a inhibitors 56 could be a potential and potent approach to enhance local bone formation for treatment of bone defects and fractures particularly in age‐related osteoporotic patients.

A complex relationship between miR‐99a and some other miRs exists. There are three miR clusters with high homology: miR‐99a/let‐7c/miR‐125b; miR‐99b/let‐7e/miR‐125a; miR‐100/let‐7a‐2/miR‐125b 57. We believe that many miRNAs co‐ordinate with each other for the regulation of osteogenic differentiation, which has been proved by previous studies 58, 59, 60, 61, 62. We may consider the complex regulatory network involving other miRs in our future study.

## Conclusions

In conclusion, our findings suggest that miR‐99a plays an important role in regulating the KDM6B levels during osteogenic differentiation. Tissue‐specific inhibition of miR‐99a might be a potential novel therapeutic strategy for enhancing *in vivo* bone tissue regeneration.

## Author contributions

Y.T.: conception and design, data collection and assembly, data analysis and interpretation, and manuscript writing; L.Z., T.T., Y.L.: data collection and assembly, data analysis and interpretation; D.M.: language editing; Q.T., J.C.: conception and design, financial support, data analysis and interpretation, and manuscript writing.

## Conflicts of interest

The authors indicate no potential conflicts of interest.
